# Complete Genome Sequence of *Mycoplasma capricolum* subsp. *capripneumoniae* Strain 9231-Abomsa

**DOI:** 10.1128/genomeA.01067-14

**Published:** 2014-10-16

**Authors:** Virginie Dupuy, François Thiaucourt

**Affiliations:** a CIRAD, UMR CMAEE, Montpellier, France; b INRA, UMR1309 CMAEE, Montpellier, France

## Abstract

*Mycoplasma capricolum* subsp. *capripneumoniae* is the etiological agent of contagious caprine pleuropneumonia. We report here the complete and annotated genome sequence of *M. capricolum* subsp. *capripneumoniae* strain 9231-Abomsa.

## GENOME ANNOUNCEMENT

*Mycoplasma capricolum* subsp. *capripneumoniae* is the causative agent of contagious caprine pleuropneumonia, a devastating disease affecting goats and some wild ruminants included in the list of notifiable diseases of the World Organisation for Animal Health (OIE) (http://www.oie.int/fileadmin/Home/eng/Health_standards/tahm/2.07.06_CCPP.pdf). In recent years, with the improvement of sequencing technologies, several complete mycoplasma genomes have become publicly available. However, a whole-genome sequence of the serious pathogen *M. capricolum* subsp. *capripneumoniae* is not yet available; only a scaffold sequence of an isolate from China has been published (1). We report here the genome sequence of *M. capricolum* subsp. *capripneumoniae* strain 9231-Abomsa, isolated in Ethiopia in 1982 (2). This constitutes the first complete circularized genome sequence of the species.

The genome sequence of strain 9231-Abomsa was obtained using a combination of 454 sequencing (GS FLX system-Roche) and Illumina HiSeq 100-bp paired-end reads. Assembly was performed using Newbler (version 1.1; 454 Life Sciences) and SeqMan NGen (version 2.0; DNAStar Lasergene, Madison, WI). The two rRNA operons were resolved by gene walking PCR. This resulted in a draft genome consisting of 5 scaffolds. This draft was then upgraded using PacBio single-molecule real-time (SMRT) sequencing technology (GATC, Konstanz, Germany). The PacBio RS sequencing data were generated by constructing an 8- to 12-kb insert library, and they were analyzed with the SMRT Analysis pipeline (PacBio DevNet; Pacific Biosciences), yielding 83✕ average genome coverage. The initial assembly was confirmed, and all gaps were resolved. Expert genome annotation was performed with MicroScope, an integrated platform for microbial genome annotation and comparative analysis (3).

The complete genome consists of a single circular chromosome of 1,017,293 bp, with an overall G+C content of 23.66%. It contains 746 coding sequences (CDSs), 248 pseudogenes, and 43 RNAs. A single CDS coded for the putative transposase OrfA of an insertion sequence similar to IS*1296*. Two putative prophage proteins were detected. No confirmed clustered regularly interspaced short palindromic repeat (CRISPR) units or integrative conjugative elements were identified. The gap that remains unresolved in the genome sequence of Chinese strain *M. capricolum* subsp. *capripneumoniae* M1601 corresponds to a region containing a cluster of variable lipoprotein genes in the 9231-Abomsa genome sequence. The two rRNA operons presented a high number of interoperon polymorphisms distributed along the entire operon sequences, including the 23S rRNA gene. This feature, already observed in the 16S rRNA gene, has been used for molecular epidemiology studies (4).

Complete circular annotated genomes are now available for all *Mycoplasma* species belonging to the *Mycoplasma mycoides* cluster (5). This provides the foundation for future studies on genome organization plasticity and comprehensive comparative genomics both intra- and interspecifically.

### Nucleotide sequence accession number.

This whole-genome sequence has been deposited in DDBJ/ENA/GenBank under the accession no. LM995445.
